# PKR-Mediated Phosphorylation of eIF2a and CHK1 Is Associated with Doxorubicin-Mediated Apoptosis in HCC1143 Triple-Negative Breast Cancer Cells

**DOI:** 10.3390/ijms232415872

**Published:** 2022-12-14

**Authors:** Sol Lee, Ha-Yeon Jee, Yoon-Gyeong Lee, Jong-Il Shin, Yong-Joon Jeon, Ji-Beom Kim, Hye-eun Seo, Ji-Yeon Lee, Kyungho Lee

**Affiliations:** 1 Department of Biological Sciences, Konkuk University, 120 Neungdong-ro, Gwangin-gu, Seoul 05029, Republic of Korea; 2 Korea Hemp Institute, Konkuk University, 120 Neungdong-ro, Gwangin-gu, Seoul 05029, Republic of Korea

**Keywords:** apoptosis, doxorubicin, ER, eIF2α, PKR, CHK1, triple-negative breast cancer

## Abstract

Triple-negative breast cancer is more aggressive than other types of breast cancer. Protein kinase R (PKR), which is activated by dsRNA, is known to play a role in doxorubicin-mediated apoptosis; however, its role in DNA damage-mediated apoptosis is not well understood. In this study, we investigated the roles of PKR and its downstream players in doxorubicin-treated HCC1143 triple-negative breast cancer cells. Doxorubicin treatment induces DNA damage and apoptosis. Interestingly, doxorubicin treatment induced the phosphorylation of eukaryotic initiation factor 2 alpha (eIF2α) via PKR, whereas the inhibition of PKR with inhibitor C16 reduced eIF2α phosphorylation. Under these conditions, doxorubicin-mediated DNA fragmentation, cell death, and poly(ADP ribose) polymerase and caspase 7 levels were recovered. In addition, phosphorylation of checkpoint kinase 1 (CHK1), which is known to be involved in doxorubicin-mediated DNA damage, was increased by doxorubicin treatment, but blocked by PKR inhibition. Protein translation was downregulated by doxorubicin treatment and upregulated by blocking PKR phosphorylation. These results suggest that PKR activation induces apoptosis by increasing the phosphorylation of eIF2α and CHK1 and decreasing the global protein translation in doxorubicin-treated HCC1143 triple-negative breast cancer cells.

## 1. Introduction

Protein kinase R/protein kinase dsRNA-dependent (PKR) is activated by dsRNA and involved in the antiviral pathway by interferons [1]. PKR plays crucial roles in various cellular processes, such as cell proliferation and stress responses [2]. It has been studied in various disease models, such as neurodegenerative disease, cancer, and leukemia models [3,4,5]. In addition to viral infection, DNA damaging agents, cytotoxic cytokines, endoplasmic reticulum (ER) stress, and growth factors increase PKR activation. Moreover, PKR activation is dependent on caspase activation during apoptosis [2,6,7].

In response to various stresses, eukaryotic initiation factor 2 alpha (eIF2α) is phosphorylated at serine 51 by one of four kinases: PKR, PKR-like ER kinase (PERK), general control non-derepressible-2, and heme-regulated inhibitor [8]. Phosphorylation of eIF2α further inhibits the translation of global proteins [9], but specific mRNAs, such as activating transcription factor 4 (ATF4), are translated. Increased ATF4 expression induces the expression of cellular homeostasis regulatory genes, such as C/EBP homologous protein (CHOP), to alleviate stress [10]. ER stress is the best-known cause of CHOP expression. CHOP is associated with apoptosis and induces the expression of pro-apoptotic genes [11].

During genotoxic stress, PKR is associated with various transcription factors, such as p53 and nuclear factor-κB [12,13]. Several studies have reported the association between PKR and p53 during cell growth [12,14]. PKR is associated with cellular signal transduction that occurs in response to certain types of DNA damage, possibly leading to p53 phosphorylation via the phosphoinositide 3-kinase (PI3K) pathway [12]. PKR activity in the nucleus has also been studied. Nuclear PKR inhibits DNA damage response and repair, and high PKR expression has been reported in acute myeloid leukemia (AML) [4,15]. Previous studies have shown that PKR plays an important role in various cellular processes.

Doxorubicin, a member of the anthracycline family, exerts significant anticancer effects in various human cancer types, including solid tumors and hematological malignancies, and is used as a major chemotherapeutic drug [16]. However, adverse side effects, such as drug resistance (chemical resistance) and cardiotoxicity, limit the use of doxorubicin for cancer treatment [17]. Several mechanisms, such as direct DNA damage, reactive oxygen species (ROS) generation, and induction of apoptosis, have been reported for the anticancer effects of doxorubicin [18,19]. Doxorubicin induces PKR activation by inhibiting the transcription of the non-coding RNA (ncRNA), nc886, as well as cytotoxicity in actively proliferating and quiescent cells [20]. Doxorubicin exerts anticancer effects through various intracellular mechanisms, and many studies have focused on overcoming its side effects. Understanding its specific action mechanism is important to increase the efficacy of doxorubicin and overcome its side effects.

Triple-negative breast cancer (TNBC) is defined as a tumor that lacks the estrogen and progesterone receptor expression and shows a lack of or decreased expression of erb-b2 receptor tyrosine kinase 2 (ERBB2, also known as HER2) [21]. TNBC is more aggressive than other breast cancer subtypes as it is difficult to treat with HER2-targeted trastuzumab, and hormone therapy is ineffective against it [22]. The treatment options for TNBC include targeted therapy and the use of cytotoxic agents [23]. Targeted therapy is a therapeutic option that targets a receptor that is specifically expressed only in cancer cells; poly(ADP ribose) polymerase (PARP) and epidermal growth factor receptor inhibitors are currently being studied for TNBC treatment [24]. In addition, cytotoxic agents, including platinum-containing agents, such as cisplatin and carboplatin, commonly induce cellular DNA damage [25]. Other cytotoxic agents, such as taxanes and anthracycline, have very short-term effects on TNBC compared to other breast cancer subtypes. However, because cytotoxic agents are cytotoxic not only to cancerous cells but also to normal cells, the overall 5-year survival rate is worse in TNBC than in other breast cancer subtypes [25]. To overcome the side effects of drugs used to treat TNBC, it is necessary to understand the mechanism of action of these cytotoxic agents. Therefore, in this study, the role of PKR activation in doxorubicin-mediated apoptosis in TNBC was investigated.

## 2. Results

### 2.1. Doxorubicin Induces DNA Damage and Apoptosis in HCC1143 TNBC Cells

**Figure 1:**
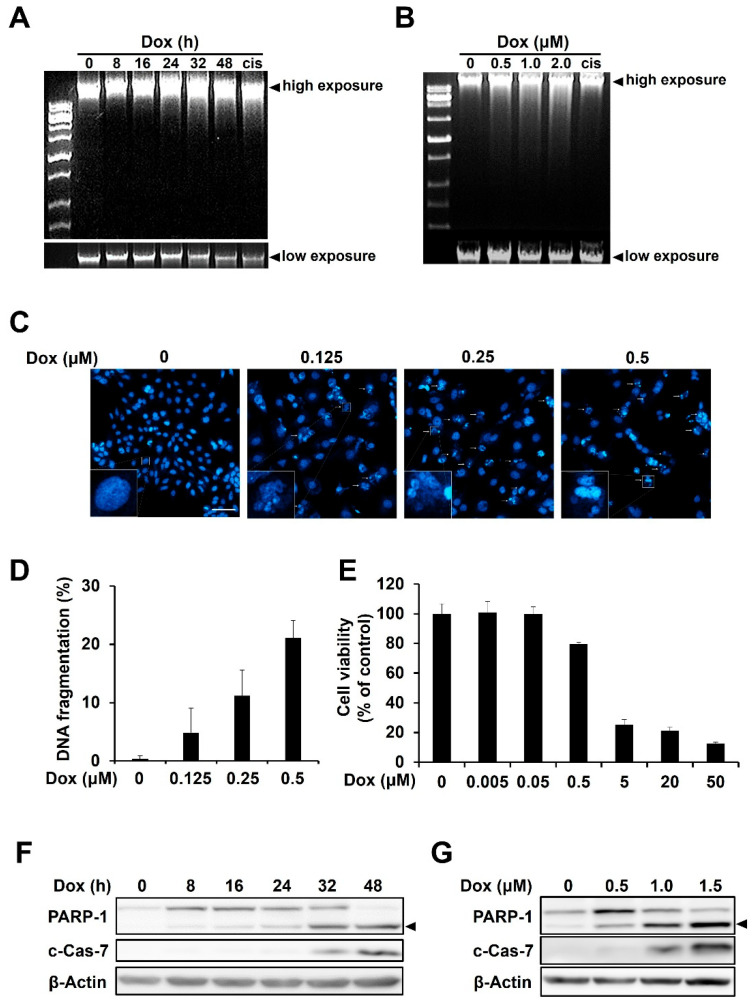
Doxorubicin induces DNA damage and apoptosis in HCC1143 triple-negative breast cancer cells. (**A**,**B**) Cells were treated with doxorubicin for the indicated time period or dose. DNA was extracted from cells and electrophoresed on agarose gels. Cisplatin (cis) was used as a positive control. (**C**,**D**) Cells were treated with the indicated concentrations of doxorubicin for 48 h and stained with Hoechst 33342, at room temperature, for 10 min. Nuclear morphological changes were observed using a confocal laser scanning microscope (magnification 100×). Fragmented nuclei are indicated by arrows, and the lower left squares are enlarged representative images (**C**). Scale bar is 100 μm. Fragmented nuclei were counted using a confocal laser scanning microscope (**D**). (**E**). HCC1143 cells were treated with doxorubicin at various concentrations for 48 h and MTT assay was performed to measure viability of cells. (**F**,**G**) Cells were treated with 1.5 μM doxorubicin for the indicated time period (**F**) or with increasing concentrations of doxorubicin for 48 h. (**G**) Immunoblotting analyses were conducted using specific antibodies against PARP, cleaved form of caspase-7 (c-Cas-7), and β-actin. Arrowhead indicates cleaved form of PARP-1.

Doxorubicin, widely known as an anticancer agent, induces apoptosis by directly inducing DNA damage [18]. First, we investigated whether doxorubicin induced DNA damage and apoptosis in TNBC cells. Cell viability was investigated with three types of TNBCs (HCC1143, MDA-MB231, and MDA-MB468) in our laboratory. As a result, HCC1143 cells showed the lowest sensitivity to doxorubicin treatment (Figure S1). Therefore, the role of PKR activation in apoptosis induced by doxorubicin was studied using HCC1143 cells thereafter. When HCC1143 cells were treated with doxorubicin, fragmented DNA increased in a time- and dose-dependent manner, as measured by agarose gel electrophoresis (Figure 1A,B). Nuclear morphological changes observed using Hoechst 33342 staining in doxorubicin-treated cells revealed that doxorubicin significantly induced nuclear condensation and increased the rate of DNA fragmentation (Figure 1C,D). When HCC1143 cells were treated with doxorubicin at various concentrations, including subtoxic concentrations, cell viability was not changed at 0.05 μM or less, but significantly decreased at 0.5 μM or more in a dose-dependent manner (Figure 1E). In addition, doxorubicin induced the activation of apoptosis markers, such as cleaved forms of PARP and caspase-7, in a time- and dose-dependent manner (Figure 1F,G). These results indicate that doxorubicin induces DNA damage and apoptosis in HCC1143 cells. HCC1143 cells were treated with 1.5 μM doxorubicin for 24 h, and then the cell cycle was measured using PI staining. Cell cycle arrest was induced as the S phase increased in doxorubicin-treated HCC1143 (Figure S2).

### 2.2. Doxorubicin-Mediated Activation of PKR, but Not PERK, Is Responsible for eIF2α Phosphorylation in HCC1143 Cells

**Figure 2:**
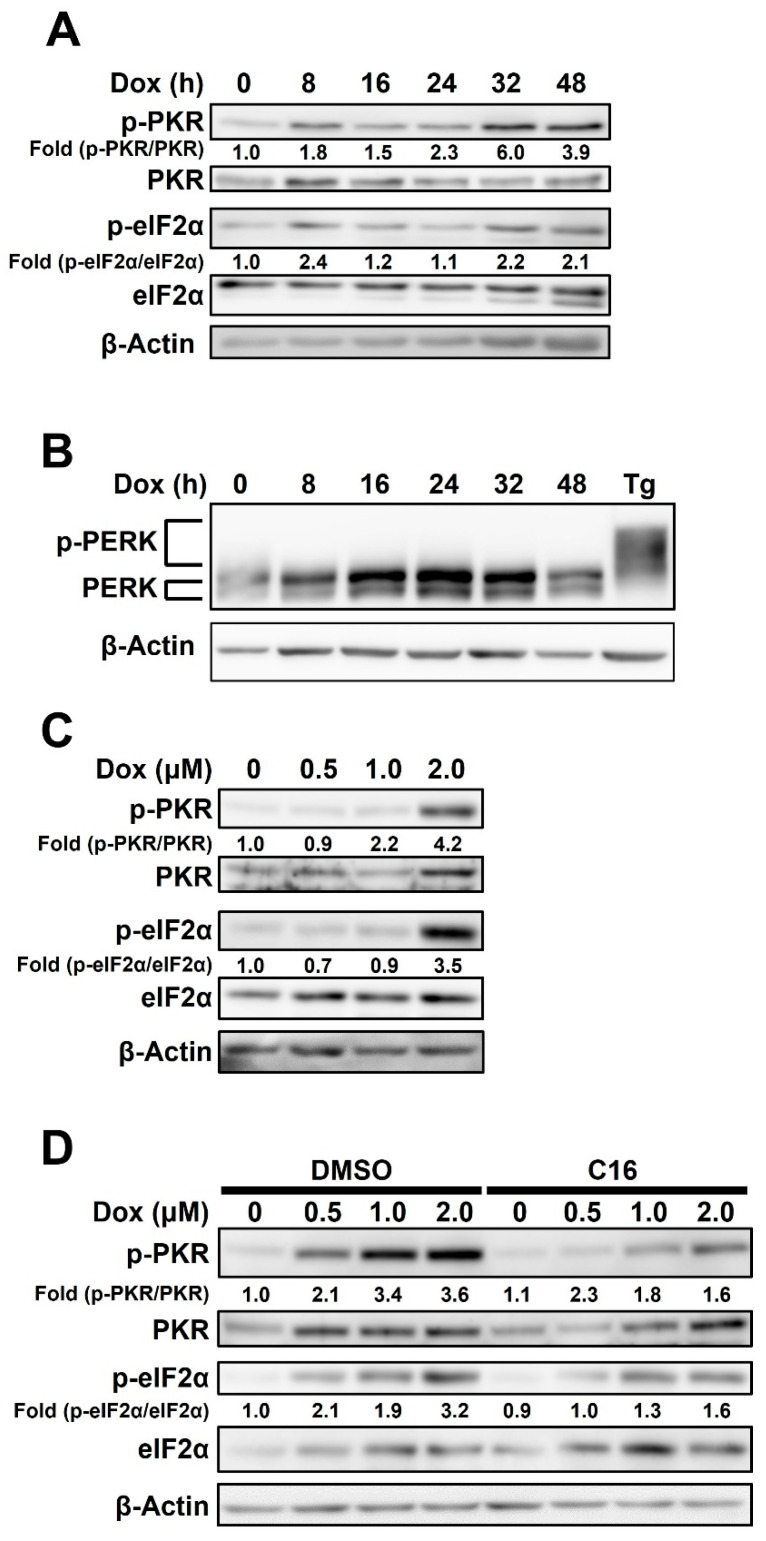
Doxorubicin-mediated activation of protein kinase R (PKR), but not PKR-like ER kinase (PERK), is responsible for eukaryotic initiation factor 2 alpha (eIF2α) phosphorylation in HCC1143 cells. (**A**,**B**) Cells were treated with 2 μM doxorubicin for the indicated time period and analyzed via immunoblotting using antibodies against phosphorylated form of eIF2α (p-eIF2α), eIF2α, phosphorylated form of PKR (p-PKR), PKR, PERK, and β-Actin. Tg was used as a positive control. Fold change is the ratios of p-PKR to PKR and p-eIF2α to eIF2α. (**C**) Cells were treated with the indicated concentrations of doxorubicin for 32 h and analyzed via immunoblotting using specific antibodies against p-PKR, PKR, p-eIF2α, eIF2α, and β-Actin. (**D**) Cells pretreated with or without 0.5 μM C16 for 1 h were treated with the indicated concentrations of doxorubicin for 32 h and analyzed via immunoblotting using specific antibodies against p-PKR, PKR, p-eIF2α, eIF2α, and β-Actin. Fold change is the ratios of p-PKR to PKR and p-eIF2α to eIF2α.

Since doxorubicin promotes PKR-mediated cell death in mouse embryonic fibroblasts [26] and regulates PKR–eIF2α signaling in breast cancer cells [3], we tested the effects of PKR on doxorubicin-treated HCC1143 cells. We tested whether doxorubicin treatment induced PKR phosphorylation in HCC1143 cells and found that PKR phosphorylation was increased by doxorubicin treatment in a time-dependent manner, peaking at 32 h after treatment (Figure 2A). Under the same conditions, eIF2α phosphorylation was only slightly increased. Similarly, the treatment of HCC1143 cells with thapsigargin increased phosphorylation of PKR (Figure S3). PERK, a kinase that induces eIF2α phosphorylation, did not show any tendency to be phosphorylated by doxorubicin (Figure 2B). Phosphorylation of PERK was significantly increased in the positive control group, in which HCC1143 cells were treated with thapsigargin (Tg) for 24 h (Figure 2B). When HCC1143 cells were treated with various concentrations of doxorubicin for 32 h, PKR phosphorylation was increased in a dose-dependent manner (Figure 2C). In addition, when HCC1143 cells pretreated with the PKR inhibitor, C16, for 1 h were treated with doxorubicin for 32 h, phosphorylation of both PKR and eIF2α decreased (Figure 2D). These results suggest that doxorubicin induces PKR phosphorylation, and doxorubicin-mediated PKR phosphorylation is associated with eIF2α activation in HCC1143 cells.

### 2.3. Inhibition of PKR Reduces the Doxorubicin-Mediated Apoptosis of HCC1143 Cells

**Figure 3:**
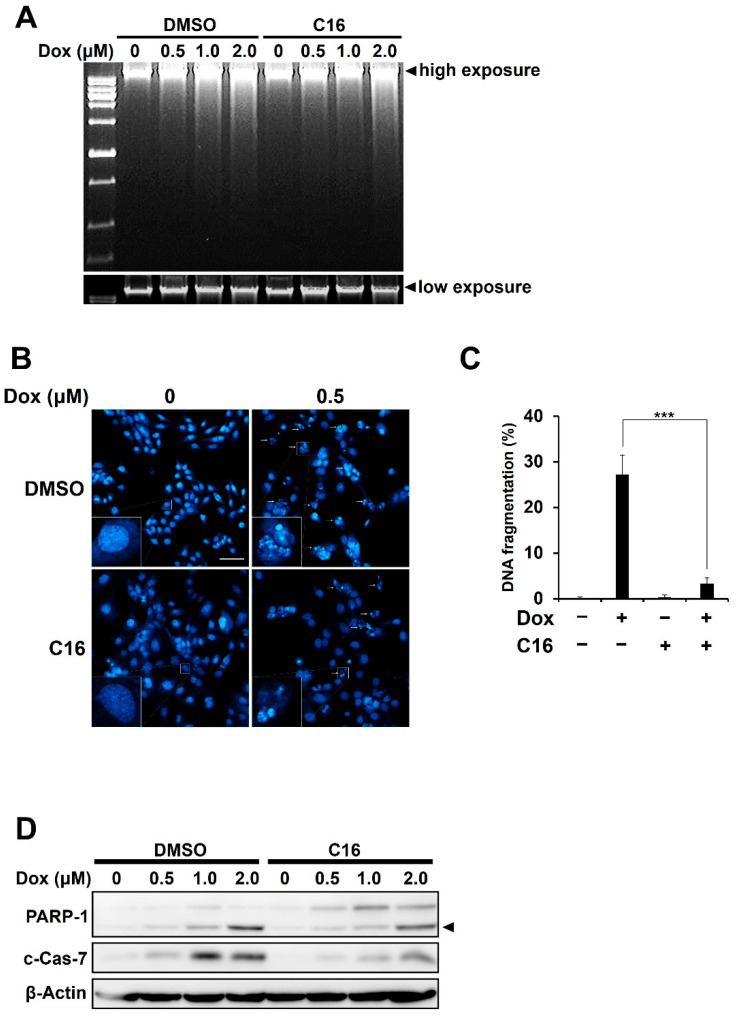
Inhibition of PKR reduces the doxorubicin-mediated apoptosis of HCC1143 cells. (**A**) Cells pretreated with or without 0.5 μM C16 for 1 h were treated with the indicated concentrations of doxorubicin for 48 h. DNA extracted from the cells was electrophoresed on agarose gels. (**B**,**C**) Cells pretreated with or without 0.5 μM C16 for 1 h were treated with 0.5 μM doxorubicin for 48 h and stained with Hoechst 33342, at room temperature, for 10 min. Nuclear morphological changes were observed using a confocal laser scanning microscope (magnification 100×). Scale bar is 100 μm. Lower-left squares are enlarged representative images (**B**). In each group, fragmented nuclei were counted using a confocal laser scanning microscope. Statistical significance was analyzed via the Student’s *t*-test (*** *p* < 0.001) (**C**). (**D**) Cells pretreated with or without 0.5 μM C16 for 1 h were treated with the indicated concentrations of doxorubicin for 32 h and analyzed via immunoblotting using specific antibodies against PARP, c-Cas-7, and β-actin. Arrowhead indicates cleaved form of PARP-1.

Since we found that doxorubicin induces eIF2α phosphorylation via PKR (Figure 2), we investigated whether PKR phosphorylation is associated with doxorubicin-mediated DNA damage. HCC1143 cells pretreated with C16 for 1 h were treated with various concentrations of doxorubicin for 48 h, and DNA damage was measured. As shown in Figure 3A, pretreatment with C16 attenuated the smear pattern of DNA fragmentation induced by doxorubicin treatment. Next, we examined the role of PKR in doxorubicin-mediated apoptosis. Cells pretreated with C16 for 1 h were treated with doxorubicin (0.5 μM) for 48 h, and nuclear morphology changes were observed using Hoechst 33342 staining. PKR inhibition reduced nuclear condensation (Figure 3B) and restored DNA fragmentation (Figure 3C). When cells pretreated with C16 for 1 h were treated with various concentrations of doxorubicin for 32 h and immunoblotting analyses were performed, the levels of cleaved forms of PARP and caspase-7 were significantly reduced (Figure 3D). These results suggest that PKR is associated with DOX-induced DNA damage and apoptosis in HCC1143 cells.

### 2.4. Doxorubicin-Induced PKR-Mediated Apoptosis Is Caused by eIF2α/CHOP Signaling and CHK1 Phosphorylation in HCC1143 Cells

**Figure 4:**
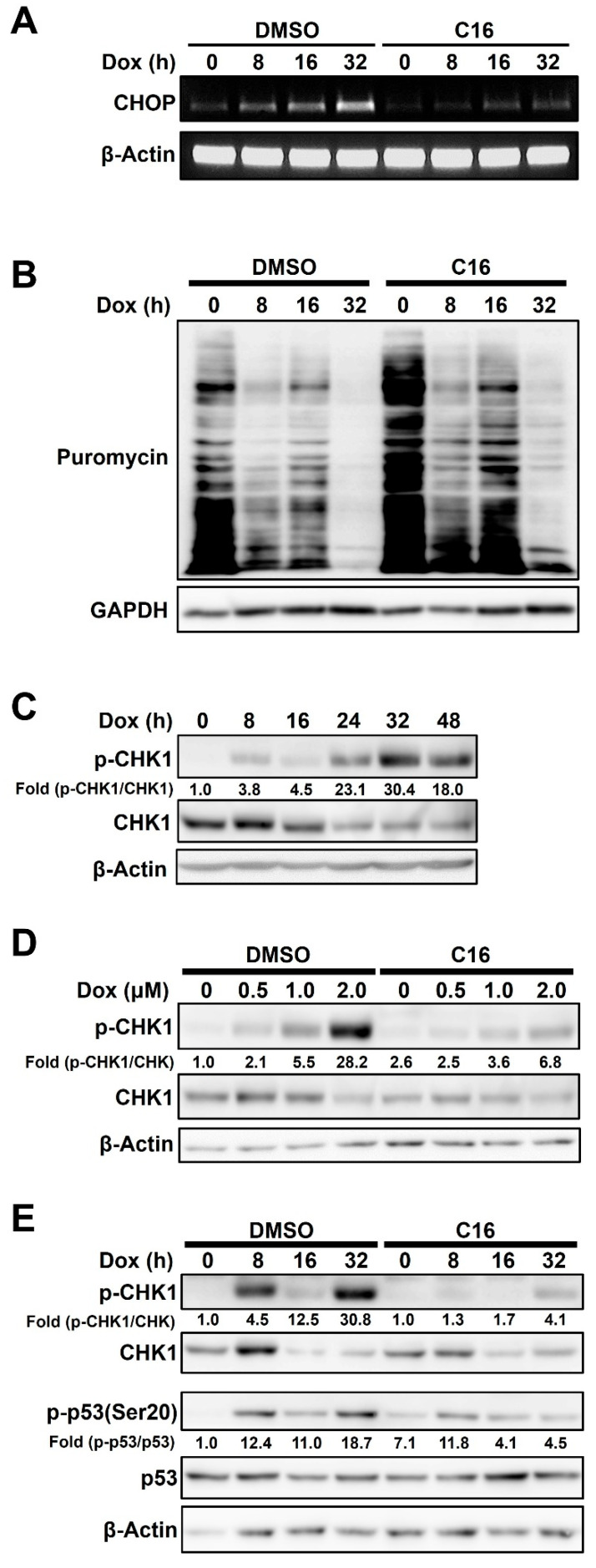
Doxorubicin-induced PKR-mediated apoptosis is caused by eIF2α/CHOP signaling and checkpoint kinase 1 (CHK1) phosphorylation in HCC1143 cells. (**A**,**B**) Cells pretreated with or without 0.5 μM C16 for 1 h were treated with 2 μM doxorubicin for the indicated time period. RT-PCR was performed using specific primers for C/EBP homologous protein (CHOP) and β-actin (**A**). Cells were treated with 10 μg/mL puromycin for 10 min. Cell lysates were subjected to immunoblotting analysis using specific antibodies against puromycin and glyceraldehyde 3-phosphate dehydrogenase (GAPDH) (**B**). (**C**) Cells were treated with 2 μM doxorubicin for the indicated time period and analyzed via immunoblotting using specific antibodies against CHK1, phosphorylated form of CHK1 (p-CHK1), and β-Actin. (**D**,**E**) Cells pretreated with or without 0.5 μM C16 for 1 h were treated with the indicated concentrations of doxorubicin for 32 h (**D**) or 2 μM doxorubicin for the indicated time period (**E**) and analyzed via immunoblotting using specific antibodies against p-CHK1, CHK1, p-p53, p53, and β-Actin. Fold change is the ratio of p-CHK1 to CHK1 (**D**) and the ratios of p-CHK1 to CHK1 and p-p53 to p53 (**E**).

Since our data showed that PKR inhibition reduced doxorubicin-mediated apoptosis in HCC1143 cells, we investigated the signaling pathways involved in this process. PKR plays important roles in apoptosis and sensitivity to doxorubicin [3,7,27]. CHOP, a downstream target of eIF2α, plays an important role in both ER stress-dependent and -independent apoptosis [11,28]. Therefore, we investigated whether PKR-mediated eIF2α phosphorylation was associated with doxorubicin-mediated apoptosis. HCC1143 cells pretreated with or without C16 for 1 h were treated with 2 μM doxorubicin for different time periods, and the expression levels of CHOP were measured via reverse transcription-polymerase chain reaction (RT-PCR). CHOP mRNA expression was increased in a time-dependent manner by doxorubicin treatment, and this effect was reduced by PKR inhibition (Figure 4A). These results indicated that PKR induces apoptosis via CHOP expression in doxorubicin-treated cells. Upon doxorubicin treatment, the increase in CHOP protein paralleled that of eIF2α phosphorylation and ATF4 (Figure S4). Next, we investigated the effect of PKR-mediated eIF2α phosphorylation on translation. Phosphorylation of eIF2α inhibits the global protein translation [29,30]. Based on this, doxorubicin-treated HCC1143 cells were treated with 10 μg/mL puromycin for the incorporation assay and analyzed via immunoblotting. As shown in Figure 4B, doxorubicin treatment decreased protein synthesis in a time-dependent manner, and inhibition of PKR by pretreatment with C16 increased protein synthesis compared to that in the dimethyl sulfoxide-treated control. Next, we investigated the role of PKR in doxorubicin-induced DNA damage response. Increased PKR expression promotes genome instability in acute leukemia [4], and doxorubicin induces the phosphorylation of checkpoint kinase 1 (CHK1), which functions in the checkpoint response process. When HCC1143 cells were treated with 2 μM doxorubicin for different time periods and analyzed via immunoblotting, phosphorylation of CHK1 was found to increase in a time-dependent manner, peaking 32 h after treatment (Figure 4C). The coincidence of the peak phosphorylation points of PKR and CHK1 after doxorubicin treatment suggests that PKR plays an important role in CHK1 phosphorylation in doxorubicin-treated HCC1143 cells (Figure 4C). To further confirm the role of PKR in the phosphorylation of CHK1, HCC1143 cells pretreated with or without C16 for 1 h were treated with various concentrations of doxorubicin for 32 h. The phosphorylation of CHK1 was clearly reduced by C16 treatment (Figure 4D), suggesting that PKR is involved in CHK1-mediated DNA damage in doxorubicin-treated HCC1143 cells. Tumor suppressor p53 plays a pivotal role in DNA damage response signaling [31], and PKR is involved in p53 activity under genotoxic stress conditions [12,32]. Therefore, we investigated whether doxorubicin-activated PKR modulated p53 activity in HCC1143 cells. When cells pretreated with or without C16 for 1 h were treated with 2 μM doxorubicin for different time periods, doxorubicin alone increased the phosphorylation of p53(Ser20), whereas the combined treatment with doxorubicin and C16 decreased this effect (Figure 4E). As shown in Figure 4D, CHK1 phosphorylation was reduced by PKR inhibition in doxorubicin-treated HCC1143 cells (Figure 4E).

## 3. Discussion

The action mechanisms of cytotoxic agents in TNBC have been studied for a long time [33]. Doxorubicin, an anthracycline-based drug, has been used for the treatment of various solid cancers, and its action mechanism has been investigated in several studies to overcome its side effects [16,34]. In this study, we investigated the role of PKR activation in DOX-mediated apoptosis in TNBC. Doxorubicin treatment induced apoptosis, as indicated by increased DNA fragmentation and levels of apoptosis markers (Figure 1). In addition, PKR was phosphorylated by doxorubicin treatment in a time-dependent manner. In contrast, PERK, which is known to phosphorylate eIF2α and regulate the ROS-induced antioxidant gene expression under ER stress [35,36], was not phosphorylated in doxorubicin-treated HCC1143 cells (Figure 2). Previously, we investigated whether PERK phosphorylated eIF2α in doxorubicin-treated HCC1143 cells. When HCC1143 cells pretreated with the PERK inhibitor, GSK2606414, were treated with doxorubicin, eIF2α phosphorylation was unexpectedly increased, and the expression levels of the antioxidant genes, such as heme oxygenase-1, glutathione-disulfide reductase, NAD(P)H quinone oxidoreductase-1, and glutathione peroxidase-1, were decreased. Moreover, when PERK was inhibited by GSK2606414, doxorubicin-mediated apoptosis was increased in HCC1143 cells. Thus, upregulation of eIF2α phosphorylation and downregulation of antioxidant gene expression by a PERK inhibitor appear to reduce doxorubicin resistance in HCC1143 cells. In contrast, simultaneous treatment with a PKR inhibitor and doxorubicin reduced the apoptosis of cells (Figure 3), suggesting that induction of eIF2α phosphorylation by PKR, but not PERK, is responsible for the apoptosis of doxorubicin-treated HCC1143 cells.

Previously, it was reported that doxorubicin induces apoptosis via PKR and that phosphorylation of eIF2α by PKR exhibits a cytoprotective effect [26]. However, in our study, inhibition of PKR using the PKR inhibitor, C16, in doxorubicin-treated HCC1143 cells resulted in the decreased phosphorylation of eIF2α and expression levels of apoptosis markers (Figure 2D and Figure 3D), suggesting that PKR-mediated phosphorylation of eIF2α plays a positive role in doxorubicin-mediated apoptosis. Next, we compared protein translation, a direct target of eIF2α phosphorylation. When cells were treated with doxorubicin alone, eIF2α phosphorylation and translational inhibition occurred simultaneously. In contrast, when PKR was inhibited by C16 treatment, eIF2α phosphorylation was reduced and global protein translation was increased (Figure 2D and Figure 4B). Therefore, it can be concluded that eIF2α phosphorylation and protein synthesis are mainly regulated by PKR under doxorubicin treatment conditions. Interestingly, when cells were treated with C16 without doxorubicin, protein translation was increased compared to the DMSO-treated control, suggesting that phosphorylation of eIF2α is regulated by PKR even under normal growth conditions in HCC1143 cells (Figure 4B). Given these results and previous reports that eIF2α phosphorylation is upregulated in the neoplastic cells of melanoma, colon cancer, Hodgkin’s lymphoma, and TNBC [37], PKR-mediated eIF2α phosphorylation can potentially serve as a marker for TNBC.

CHOP is a downstream target whose expression is regulated by eIF2α phosphorylation. CHOP is associated with apoptosis and induces the expression of pro-apoptotic genes [11,38]. It also inhibits Bcl-2 expression, depletes cellular glutathione, and induces production of reactive oxygen species [39]. As shown in Figure 4A, the level of CHOP mRNA increased with increasing doxorubicin treatment time but decreased with simultaneous treatment with C16 and doxorubicin. These results indicate that doxorubicin induces apoptosis by inducing eIF2α phosphorylation via PKR, inhibiting protein translation, and upregulating CHOP expression.

Doxorubicin induces DNA damage via DNA adduct formation, topoisomerase II poisoning and intercalation [40]. Interestingly, CHK1 phosphorylation was induced by doxorubicin treatment and decreased by PKR inhibition (Figure 4D,E). In general, phosphorylation of CHK1 is regulated by ATR and activated by UV-induced DNA damage and single-strand DNA breaks [41,42]. Although CHK1 was reported to be important for doxorubicin-induced cell cycle arrest, the relationship between PKR and CHK1 phosphorylation remains unclear [43]. According to this report and our results, there appears to be a link between doxorubicin-mediated PKR activation and CHK1 phosphorylation.

During DNA damage, the N-terminus of p53 is phosphorylated at several serine residues, including Ser20, to regulate protein stability and function [44]. p53, a representative DNA damage response factor, plays an important role in improving apoptosis in response to DNA damage stress, rather than DNA replication stress, caused by doxorubicin in prostate cancer [45]. In addition, activation of p53 by genotoxic stress induces PKR expression via the interferon-sensitive response element, leading to translational inhibition and apoptosis in cells [46]. In our study, p53(Ser20) phosphorylation was increased by doxorubicin treatment alone in TNBC cells. Co-treatment with C16 and doxorubicin reduced the phosphorylation of p53 (Figure 4E). Therefore, it can be concluded that PKR partially affects the activation of CHK1 and p53 during DNA damage.

The mechanism by which doxorubicin, which directly damages DNA, activates PKR remains ambiguous. According to a recent report, doxorubicin inhibits RNA polymerase III, thereby reducing the transcription of nc886, which inhibits PKR, resulting in PKR-mediated apoptosis [20]. In addition, PKR plays important roles in the nucleus and cytoplasm. Nuclear PKR inhibits the DNA damage response and DNA repair signaling. PKR expression is high under normal growth conditions in AML, and upregulated PKR further promotes genomic instability [4]. In a previous report on the role of PKR in doxorubicin-treated cells, doxorubicin induced cell apoptosis via PKR-mediated phosphorylation of c-jun N-terminal kinase, and phosphorylation of eIF2α had a cytoprotective effect [26]. The results of this study revealed that PKR-mediated phosphorylation of eIF2α appears to induce apoptosis by inhibiting the translation of pro-survival proteins and upregulating the expression of CHOP in doxorubicin-treated HCC1143 cells. CHK1 is associated with DNA damage induced by doxorubicin [43,47], and doxorubicin appears to be involved in the phosphorylation of CHK1 and PKR-mediated phosphorylation of eIF2α.

We searched published data sets and published articles to evaluate the relationship between PKR expression and TNBC clinicopathological parameters in cancer patients and TNBC breast cancer cells. According to The Human Protein Atlas (https://www.proteinatlas.org/ (accessed on 4 December 2022)), the 12-year survival rate for patients with invasive breast cancer with low PKR expression is approximately 70%, whereas the 12-year survival rate for patients with high PKR expression is approximately 30%. This trend is also evident in OncoDB (https://oncodb.org/index.html (accessed on 4 December 2022)), where the 12-year survival rate for breast cancer patients with low PKR expression is approximately 50%, whereas the 12-year survival rate for patients with high PKR expression is approximately 30%. By analyzing The Cancer Genome Atlas (TCGA) data set, Kung et al. showed that PKR expression is higher in TNBC samples compared to non-TNBC samples, which is consistent with RNA-seq data for breast cancer cell lines [48]. These suggest that high expression of PKR is associated with malignancy of TNBC cells.

Summarizing all the results, the conclusion of this paper is as follows. Phosphorylation of eIF2α and CHK1 induces apoptosis in Dox-treated TNBC. PERK plays an important role in eIF2α phosphorylation in many cells, but PKR plays a major role in eIF2α phosphorylation and CHK1 phosphorylation in doxorubicin-treated TNBC. The results of this study suggest that modulation of PKR activation during doxorubicin-mediated apoptosis can serve as a therapeutic target in TNBC. As PKR is involved in various cellular responses, further studies are required to establish the mechanism of doxorubicin-mediated PKR activation under various conditions.

## 4. Materials and Methods

### 4.1. Reagents

Doxorubicin, thapsigargin, poly(I:C), and puromycin were purchased from Sigma-Aldrich (St. Louis, MO, USA). PKR inhibitor, C16, was purchased from Calbiochem (La Jolla, CA, USA). Anti-phospho eIF2α (9721), anti-eIF2α (9722), anti-PERK (3192), anti-phospho p53 (Ser20, 9287), anti-CHOP (2895), and horseradish peroxidase-conjugated antibodies (Anti-Rabbit IgG, 7074; Anti-Mouse IgG, 7076) were purchased from Cell Signaling Technology (Beverly, MA, USA). Anti-PARP-1 (sc-7150), anti-caspase-7 (sc-28295), anti-β-actin (sc-47778), anti-GAPDH (sc-25778), anti-phospho CHK1 (sc-17922), anti-CHK1 (sc-8408), anti-PKR (sc-6282), anti-p53 antibodies (sc-17846), and anti-ATF4(sc-390063) were purchased from Santa Cruz Biotechnology (Santa Cruz, CA, USA). Anti-phospho-PKR antibody (ab81303) was purchased from Abcam (Cambridge, UK). Hoechst 33342 was purchased from Thermo Fisher Scientific (Waltham, MA, USA).

### 4.2. Cell Culture

HCC1143, MDA-MB231, and MDA-MB468 cells were purchased from the Korean Cell Line Bank (Seoul, Republic of Korea). Cells were cultured in the Roswell Park Memorial Institute-1640 medium (Hyclone, Logan, UT, USA), containing 10% heat-inactivated fetal bovine serum (Biowest, Nuaille, France) and 1% penicillin/streptomycin mixture (Gibco), under a humidified atmosphere of 5% CO_2_ at 37 °C. Trypsin-EDTA and penicillin/streptomycin were purchased from Gibco BRL (Carlsbad, CA, USA).

### 4.3. Measurement of Apoptosis Using Microscopy

HCC1143 cells were cultured on coverslips in a 6-well cell culture plate. After 16 h, the cells were treated with doxorubicin at various concentrations with or without a PKR inhibitor for 48 h. Cells were fixed in 4% paraformaldehyde for 10 min, at room temperature, washed thrice with PBS, and treated with PBS containing 0.1% Triton X-100 for 10 min. The cells were washed thrice with PBS and stained with Hoechst 33342 (1:2500) for 10 min in the dark, at room temperature. Cells were stained on coverslips and placed on a microscope glass slide. The morphology of the nucleus was observed using a super-resolution confocal laser scanning microscope (LSM 800; Carl Zeiss) at 350 nm for excitation (magnification 100×).

### 4.4. Measurement of DNA Fragmentation

Cells seeded in a 100 mm plate were incubated for 16 h prior to the experiment and treated with 2 μM doxorubicin for various time periods or with various concentrations of doxorubicin for 48 h, including PKR inhibitors, if necessary. Cells were harvested using TES lysis buffer (20 mM EDTA, 100 mM Tris-HCl [pH 8.0], and 0.8% SDS). RNase (1 mg/mL) was added to the DNA sample dissolved in TES buffer and incubated, at 37 °C. Proteinase K was added to the DNA samples containing RNase and incubated overnight, at 50 °C. DNA samples were electrophoresed at 50 V for 3 h on a 1.5% agarose gel.

### 4.5. Immunoblotting Analysis

Cells were harvested using NET lysis buffer (150 mM NaCl, 50 mM Tris-HCl [pH 7.4], 1 mM EDTA, and 1% Triton X-100) containing 1% phosphatase and protease inhibitors. Protein concentration was quantified using Bradford assay (Bio-Rad, Hercules, CA, USA). Proteins were boiled in 1× protein loading buffer (250 mM Tris-HCl [pH 6.8], 10% SDS, 50% glycerol, 0.02% bromophenol blue, and 500 mM β-mercaptoethanol) for 5 min, at 100 °C, and separated on SDS-polyacrylamide gels. Proteins were electrotransferred onto Immobilion-PSQ membranes (Millipore Corp., Bedford, MA, USA). The membranes were incubated with 5% skim milk for 2 h, at room temperature, and blotted with the indicated antibodies overnight, at 4 °C, in Tris-buffered saline containing 0.08% Tween 20 (TBST) and 1% skim milk. The membranes were then incubated with horseradish peroxidase-conjugated secondary antibodies for 2 h, at room temperature, and band signals were visualized using the LAS-3000 luminescent image analyzer (Fujifilm, Tokyo, Japan). To determine the equal loading of samples, blots were stripped in stripping buffer (100 mM β-mercaptoethanol, 2% SDS, and 62.5 mM Tris-HCl [pH 6.8]), at 60 °C, for 30 min, washed thrice for 10 min each with the TBST buffer, and re-probed with other specific antibodies.

### 4.6. RT-PCR and PCR Primers

Cells were cultured in 100 mm cell culture dishes. Total RNA was extracted using RNAiso Plus (TAKARA, Japan), according to the manufacturer’s protocol. Total RNA (2 μg) was used to synthesize cDNA using Improme II (Promega, Madison, WI, USA), according to the manufacturer’s protocol. PCR was performed to amplify specific genes using FIREPol DNA polymerase (Solis BioDyne, Estonia), according to the manufacturer’s instructions. Primer sequences were prepared to amplify specific genes as follows: human CHOP, forward primer 5′-TGGAAGCCTGGTATGAGGAC-3′ and reverse primer 5′-GTTCTTTCTCCTTCATGCGC-3′; human β-actin, forward primer 5′-CATGTACGTTGCTATCCAGGC-3′ and reverse primer 5′-CTCCTTAATGTCACGCACGAT-3′. PCR products were electrophoresed on 1% agarose gel for 20 min.

### 4.7. Cell Cycle Analysis

HCC1143 cells were cultured in 60 mm culture dishes. After 16 h, cells were treated with 1.5 μM doxorubicin for 24 h. Adherent and floating cells were pooled in microcentrifuge tubes, pelleted by centrifugation, washed with PBS, and fixed overnight in cold 70% ethanol at −20 °C. Cells were washed, re-suspended in 1 mL propidium iodide (PI) solution containing 20 μg/mL RNase A and 100 μg/mL PI, incubated for 30 min, at 37 °C, and assayed using a Becton Dickinson Flow Cytometer (Becton-Dickinson Biosciences, San Jose, CA, USA) at 488 nm. Data were analyzed with WinMDI version 2.9 software (Joe Trotter, Scripps Research Institute, La Jolla, CA, USA). The percentage of DNA-damaged cells was calculated as the ratios of G0/G1 phase cells and S phase cells to the total cell population.

### 4.8. Statistical Analysis

Values in the figures are expressed as the mean ± standard deviation. Figures are representative of three or more experiments. Statistical analysis of data between the control and experimental groups was performed using the Student’s *t*-test. Values of *p* < 0.05 were considered to be statistically significant.

## 5. Conclusions

- Doxorubicin-mediated activation of PKR, but not PERK, is responsible for eIF2α phosphorylation in HCC1143 triple-negative breast cancer (TNBC) cells.
- Inhibition of PKR reduces the doxorubicin-mediated apoptosis of HCC1143 TNBC cells.
- Doxorubicin-induced PKR-mediated apoptosis is caused by eIF2α/CHOP signaling in HCC1143 TNBC cells.
- Doxorubicin-induced PKR-mediated apoptosis is caused by CHK1 phosphorylation in HCC1143 TNBC cells.

## Supplementary Materials

The following supporting information can be downloaded at: https://www.mdpi.com/article/10.3390/ijms232415872/s1.
