# Supplementary material for: PKR-Mediated Phosphorylation of eIF2a and CHK1 Is Associated with Doxorubicin-Mediated Apoptosis in HCC1143 Triple-Negative Breast Cancer Cells
Source: Int J Mol Sci. 2022 Dec 14;23(24):15872. doi: 10.3390/ijms232415872 (PMC9779813; doi:10.3390/ijms232415872)
Supplement: Supplementary file 1 [file ijms-23-15872-s001.zip › ijms-2048109-supplementary.pdf]

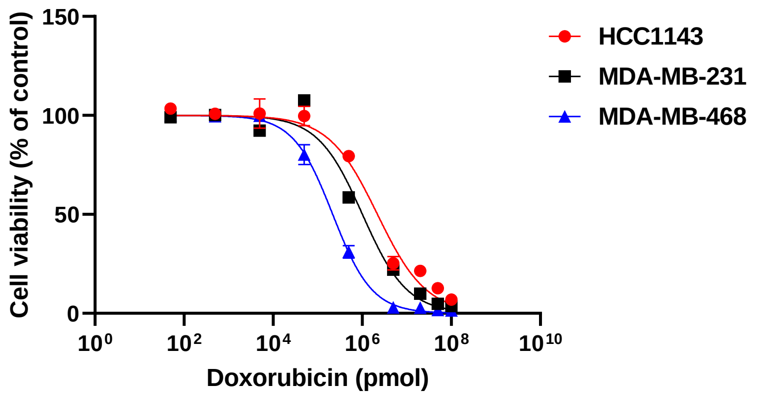

Figure S1. Sensitivity of various triple negative breast cancer cells to doxorubicin. Three types of TNBC (HCC1143, MDA-MB-231, MDA-MB-468) were treated with doxorubicin at various concentrations for 48 h and MTT assay was performed to measure cell viability.

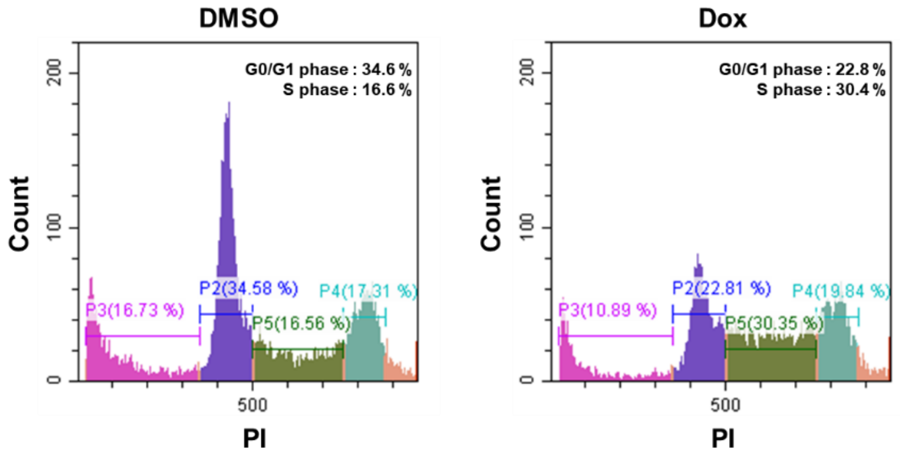

Figure S2. Doxorubicin-mediated DNA damage in HCC1143 triple-negative breast cancer cells. Cells were treated with DMSO (control) or 1.5 μM doxorubicin for 24 h, stained with PI, and subjected to cell cycle analysis. Percentages of cells counted in G0/G1 phase and S phase are indicated by bars in in the histogram.

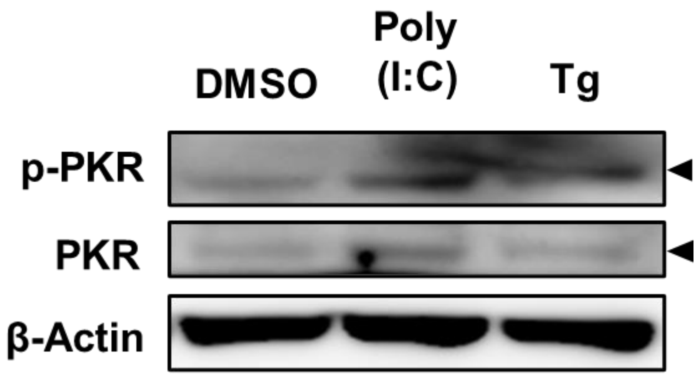

Figure S3. Activation of PKR-mediated eukaryotic initiation factor 2 alpha (eIF2α) in HCC1143 cells. Cells treated with DMSO (control), 1.5 μM doxorubicin, 20 μM Poly(I:C), and 0.2 μM Tg for 24 h were analyzed via immunoblotting using antibodies against p-PKR, PKR, and β-Actin.

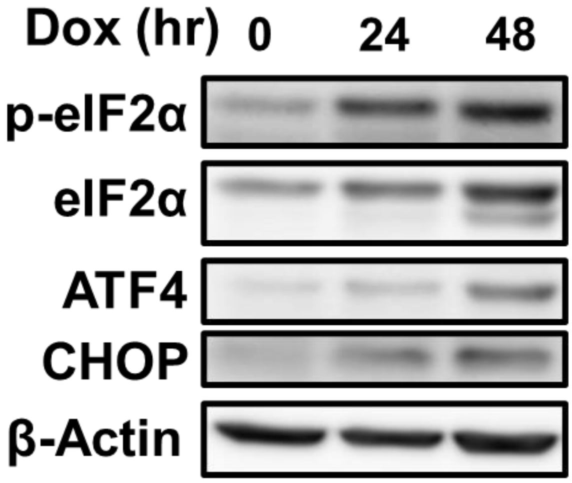

Figure S4. Doxorubicin-mediated eIF2α/ATF4/CHOP signaling in HCC1143 cells. Cells were treated with 1.5 μM doxorubicin for the indicated time period and analyzed via immunoblotting using specific antibodies against p-eIF2α, eIF2α, ATF4, CHOP, and β-Actin.
